# *Strongyloides stercoralis* Hyper infection Syndrome

**DOI:** 10.1007/s12262-020-02292-x

**Published:** 2020-05-12

**Authors:** Sampath Kumar Karanam L, Gopi Krishna Basavraj, Chaitanya Kumar Reddy Papireddy

**Affiliations:** Department of General Surgery, Narayana Medical College, Chinthareddy Palem, Nellore, Andhra Pradesh 524003 India

**Keywords:** *Strongyloides stercoralis*, Strongyloidiasis, Hyper infection syndrome, Disseminated infection, Immunity

## Abstract

*Strongyloides stercoralis* is a helminth, widely distributed in tropical and subtropical countries. Its infestation in humans usually does not produce symptoms. However, in some patients, severe and life-threatening forms of this infection can occur, especially in immunocompromised individuals. Severe parasitic infection is triggered by any imbalance in the host’s immunity favouring the auto-infective cycle. This results in an increase in the intraluminal parasitic burden. In addition, tissue infestation is also very common. Clinical presentation is variable, and it is very difficult to diagnose clinically. Diagnosis requires a high index of suspicion. In some cases, the diagnosis is established only on histopathological examination of the excised tissue by the pathologist. Here, the authors report a case of an elderly male diabetic patient, who presented to the emergency department with the features of acute abdomen. On exploratory laparotomy, he was found to have the features suggestive of gangrene of small bowel. Resection of the gangrenous bowel was done, and end-to-end anastomosis was done as the rest of the bowel appeared to be normal. However, the patient died of multi-organ failure and septicaemia on the second postoperative day. The resected intestine showed tissue infestation of *Strongyloides stercoralis* on histopathological examination. In this review article, the authors summarize a case of hyper infection syndrome of strongyloidiasis and discuss the various aspects of *Strongyloides stercoralis* infection with emphasis on life cycle of the parasite and different clinical features of the disease.

## Introduction

In tropical and subtropical regions, wherever there is poor hygiene and sanitation, helminthic infection is very common [1]. Strongyloidiasis is caused by a soil dwelling nematode helminth, *Strongyloides stercoralis*. As a parasite, it resides in the small intestine of a human being. *Strongyloides stercoralis* infection was first reported in the year 1876 in French soldiers working in Vietnam [2]. In most of the cases, the intestinal strongyloidiasis does not produce any symptoms. A rare and characteristic feature of autoinfection occurs in its life cycle. As a result, whenever there is an immune imbalance between the host and the parasite, life-threatening complications like hyper infection syndrome and disseminated tissue infestation can occur [3–5]. First report of hyper infection syndrome and disseminated infection appeared in patients with immune suppression in the year 1966 [6]. In hyper infection syndrome, the extensive larval proliferation leads to systemic sepsis and multi-organ failure. Normally, the larvae are present in the traditional life cycle sites such as the skin, gastrointestinal tract or lungs. Sometimes, the larvae may be found in heart, brain, muscle, etc., and then it is known as disseminated disease. Most of the patients with hyper infection syndrome often have catastrophic clinical manifestations such as shock, disseminated intravascular coagulation, meningitis, renal failure and respiratory failure. Sometimes, the patient may present with acute abdomen as in our case. An elderly diabetic patient presented with the features of acute abdomen found to have gangrenous small bowel on exploratory laparotomy. The cause for his catastrophic presentation was found to be strongyloidiasis hyper infection syndrome, revealed on histopathological examination of the resected specimen.

This review article presents an unsuspected case of strongyloidiasis hyper infection syndrome presented as an acute abdominal catastrophe and discusses the pathogenesis and various presentations of this unusual entity.

## Case Report

**Fig. 1:**
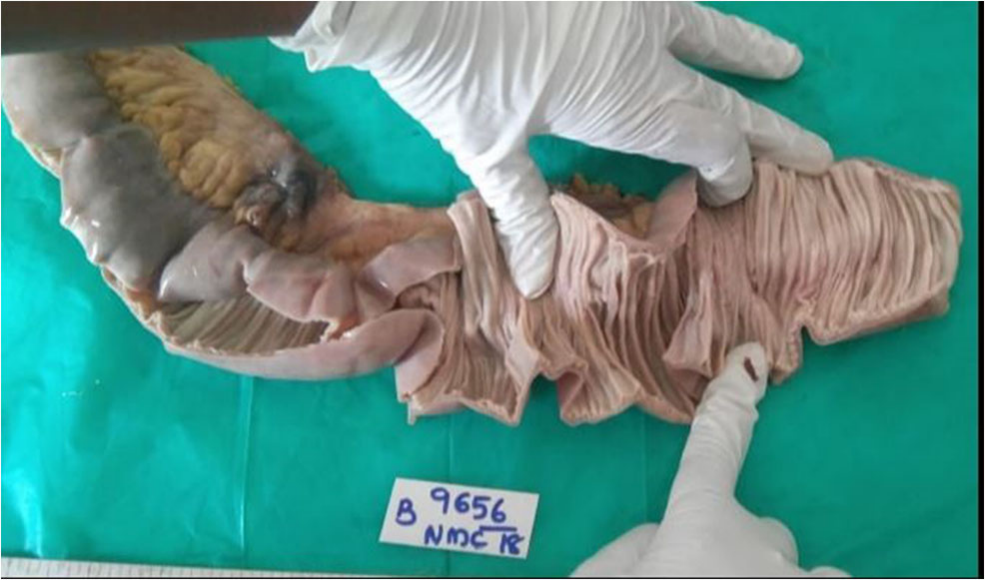
The resected jejunal loop with dusky hue patch (on the right upper corner)

A 56-year-old male, a known diabetic patient who consumed alcohol from the age of 22 years, came to the hospital with the features suggestive of peritonitis of 24-h duration. On examination, he was febrile, ill-looking patient with tachycardia and tachypnoea. Local examination of abdomen revealed distended, tender abdomen with rigidity. There was clinical evidence of free fluid in the peritoneal cavity, and bowel sounds were absent. Clinical diagnosis of peritonitis as a result of hollow viscous perforation was made. Plain radiograph of the chest and upper abdomen in erect position revealed the presence of free air under both domes of the diaphragm. Total leukocyte count was raised with eosinophilia. The blood sugar levels were raised and controlled with insulin prior to surgery. The serum creatinine was raised to 2.2 mg%. The patient was taken up for emergency exploratory laparotomy. On midline laparotomy, there was evidence of bile-stained free fluid in the peritoneal cavity. Approximately 30 cm of jejunal loop, 40 cm from the duodeno-jejunal junction was found to have multiple patches of dusky hue colour on the jejunal serosal surface. There were multiple small perforations in the same jejunal loop (Fig. 1).

Rest of the bowel was found to be normal with normal vasculature. The mesenteric vessels were found to be normal. In view of the above findings, resection of the diseased jejunal segment was done, and end-to-end anastomosis was performed. The patient was put-on broad-spectrum antibiotics along with metronidazole intravenously. Postoperatively, the investigations did not reveal any abnormality in the coagulation profile.

**Fig. 2:**
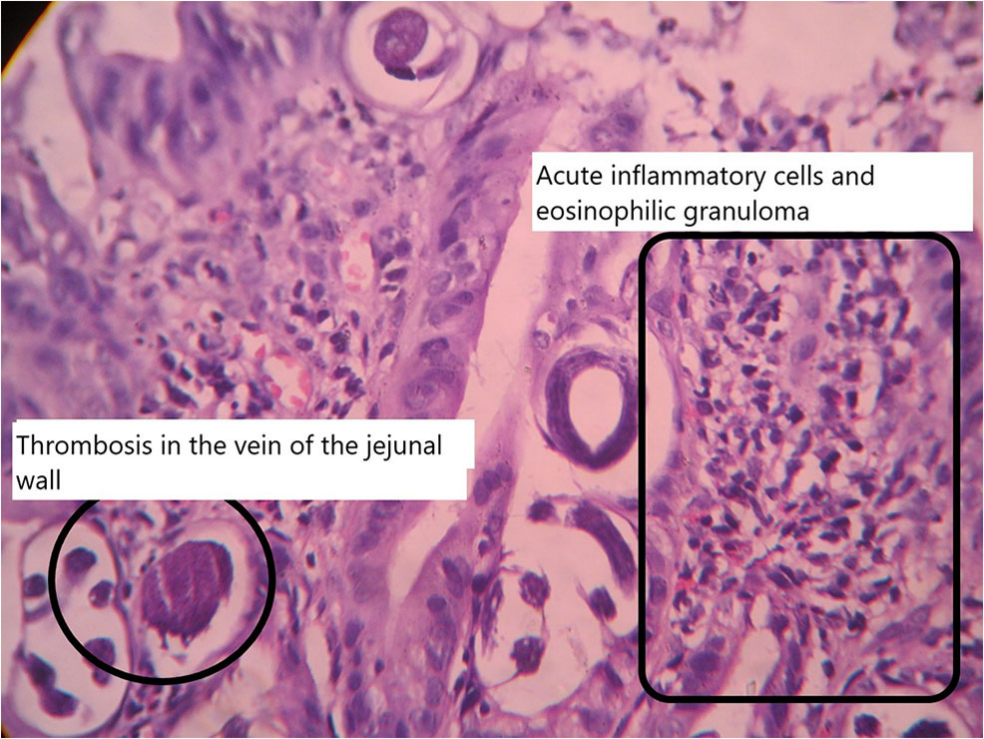
Acute inflammatory cells, eosinophilic granulomas and venous thrombosis in the jejunal wall

The immediate postoperative period was stormy. On the first postoperative day, the patient developed the features of acute respiratory distress syndrome and required mechanical ventilatory support. Very soon, he developed multi-organ failure and succumbed to death in spite of our best efforts within 24 h of surgery. Most probable cause of death was presumed to be Gram-negative bacterial septicaemia in view of presence of source in the form of hollow viscous perforation and the clinical features. The blood culture report which was collected later on did not reveal any bacterial growth. However, the histopathological report of the resected jejunal segment revealed the changes of acute inflammatory reaction in the intestinal wall with marked eosinophilic granulomas with focal areas of haemorrhagic necrosis. There was evidence of extensive local venous thrombosis (Fig. 2).

**Fig. 3:**
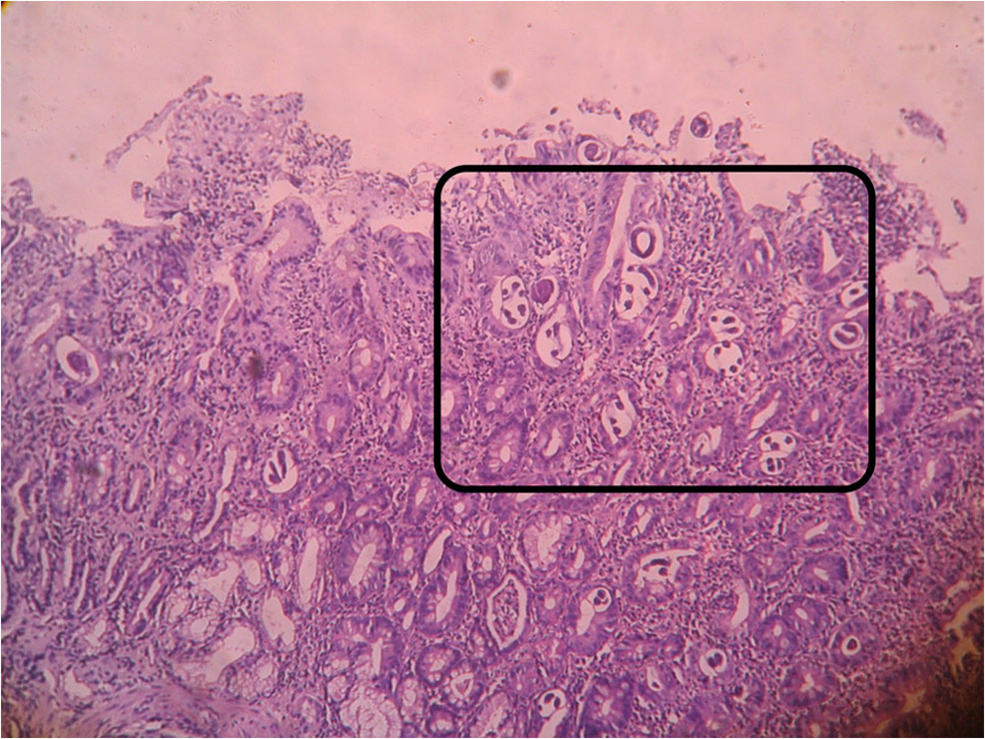
Multiple filariform larvae of *Strongyloides stercoralis* in the tissue planes of the jejunal wall.

There was evidence of filariform larvae of the parasite, *Strongyloides stercoralis* in the tissue planes of the jejunal wall (Fig. 3).

## Discussion

The strongyloidiasis is ubiquitous in tropical and subtropical regions. It is caused by *Strongyloides stercoralis*, a nematode helminthic parasite. The life cycle of *Strongyloides stercoralis* is comprised of two parts: a free-living life cycle outside of the host as rhabditiform larva in the soil and a parasitic life cycle when the rhabditiform larva penetrates the human skin. After entering the venous circulation, the larva passes through the right heart and lungs. During the maturation process, strongyloides larvae induce alveolar capillary bleeding and potent eosinophilic inflammation resulting in eosinophilic pneumonitis.

From the alveoli, larvae continue to migrate up the pulmonary tree and trachea. The cough reflex helps to push the larvae out of the bronchial tree and trachea. However, once the larvae reach the larynx, they are swallowed and travel to the stomach and small bowel. Inside the small bowel, strongyloides larvae mature into diminutive adult forms that measure approximately 220–250 μm. Adult female worms embed themselves in the mucosa of the small intestine and produce eggs via parthenogenesis. Within the intestinal lumen, the eggs hatch into non-infective rhabditiform larvae, which are excreted, along with the stool [7].

A unique feature of this parasite is its ability to cause autoinfection. In this process, the parasite does not reach the soil, but it reaches the same human host via enteral circulation (endo autoinfection) or through the penetration of perianal skin (exo autoinfection) [5]. In addition, dirty hands or food contaminated with stool can carry infective filarial larvae from the anus back to the host (faeco-oral route).

The vast majority of patients with strongyloidiasis has uncomplicated disease. Majority of patients remain asymptomatic and survive normal life undiagnosed. Amongst symptomatic patients, initial acute infection is generally manifested as serpiginous urticarial rash at the site of penetration of filariform larva usually in the leg. Some of them may also have cough, tracheal irritation mimicking bronchitis from the larval migration through the respiratory system and gastrointestinal symptoms such as nausea, vomiting, mild pain upper abdomen, bloating sensation and watery diarrhoea once the parasite reaches its normal habitat, the small intestine. Most of these patients receive symptomatic treatment and recover without the proper diagnosis of strongyloidiasis. They continue to lead normal life but still harbouring the parasite. Some of them may suffer from chronic condition of the disease in the form of intermittent attacks of dyspepsia and intermittent diarrhoea. Some of them may suffer from respiratory symptoms such as intermittent asthmatic attacks. Unless physicians have high index of suspicion, there is likelihood of misdiagnosis or no diagnosis at all. However, most of these patients show eosinophilia.

About 1.5% to 2.5% of patients harbouring the parasite, *Strongyloides stercoralis* may present with two different clinical entities known as hyper infection syndrome and disseminated strongyloidiasis [8]. In hyper infection syndrome, the classic life cycle is exaggerated, resulting in an increase in the parasitic burden as a result of autoinfection [5]. Although hyper infection can occur in any host, the incidence is more common in immunocompromised individuals. Disseminated disease is defined by the presence of parasite outside of the traditional life cycle sites, i.e. in organs other than the skin, lungs and gastrointestinal tract [9]. The parasite can be seen in organs such as liver, muscle, heart and central nervous system. When compared with hyper infection syndrome, disseminated disease occurs mainly in immunocompromised individuals such as transplant patients, patients receiving steroids or immunosuppressants or patients infected with human T cell lymphotropic virus type [10]. The clinical presentation of hyper infection syndrome is similar to that of classic strongyloidiasis, involving gastrointestinal tract and lungs, which include nausea, vomiting, diarrhoea, weight loss, abdominal pain, GI haemorrhage, subacute intestinal obstruction or eosinophilic granulomas producing enterocolitis, cough, fever and dyspnoea [11, 12]. However, due to increased parasitic burden, some of these patients often have catastrophic clinical manifestations as in our case.

In disseminated strongyloidiasis, there is widespread dissemination of larvae outside the gut and lungs, often involving the liver, brain, heart and urinary tract [5]. As expected, in disseminated infection, multiple organ systems are involved in addition to respiratory and gastrointestinal systems. The central nervous system involvement leads to headache, altered sensorium, seizures and even coma. In these compromised immunity patients, Gram-negative bacterial meningitis is common [13].

Diagnosis of strongyloidiasis hyper infection syndrome or disseminated disease is very difficult to establish and requires a high level of suspicion [14]. Most of these patients present with non-specific clinical features, and the laboratory, imaging and endoscopic findings are often non-contributory also as in our case. Moreover, most of the patients present decades later, after primary infection. However, in some cases due to the large parasitic burden, the yield in lung, bronchial and small bowel biopsies is very high, and the diagnosis is possible [11]. It can cause life-threatening infection in an immunocompromised host with 60%–85% mortality rate [15]. Steroid therapy, human T cell leukaemia, HIV infection, malignancy, chronic renal failure, diabetes mellitus, advanced age, collagen vascular disease and chronic alcoholic consumption can cause life-threatening complications in strongyloidiasis. Life-threatening gastrointestinal complications include gastrointestinal bleeding from micro aneurysms in the mesenteric vessels or infarction and perforation of the intestine from local intestinal wall venous thrombosis as in our case [16]. Sometimes, strongyloidiasis is associated with gut translocation of bacteria leading to polymicrobial bacteraemia and septicaemia [17]. Commonly associated bacteria include Gram-negative rods such as *Escherichia coli* and Gram-positive cocci such as *Streptococcus bovis*. In fact, the positive blood culture of *Streptococcus bovis* in a patient may suggest the presence of strongyloidiasis or gastrointestinal malignancy [18]. However, in our patient, blood culture did not reveal any bacterial growth though we subjected only one specimen for culture. Most probably, in our patient as a result of hyper infection syndrome, the infective filariform larvae must have penetrated into the jejunal wall layers as denoted by the presence of larvae in the resected specimen. These infective larvae must have produced eosinophilic granulomas and thrombosis of local jejunal wall veins. Once the jejunal vasculature was pathologically involved, it must have produced patches of ischaemia and multiple small perforations. At the time of laparotomy in our case, the mesenteric vasculature, both arteries and veins were found to be normal. Moreover, in our case, very limited length of small bowel was found to be affected; only 30 cm long jejunum was affected, unlike a typical mesenteric ischaemia. In addition, there must have been translocation of enteral bacteria resulting in polymicrobial septicaemia as our patient died of multi-organ failure secondary to septicaemia. This type of scenario is very common in diabetics and in people with chronic alcohol consumption as in our patient.

Sometimes, death can occur in asymptomatic strongyloides infection when exhibited to steroids or immunosuppression [19]. Multiple case reports exist of patients with fatal outcome after treatment with steroids for an unrelated condition and the pathologist later demonstrated disseminated strongyloidiasis [20]. By and large, the clinical features of strongyloidiasis vary depending on the acuity of infection and the underlying host response [21]. So, it is very important to consider the possibility of strongyloidiasis in any immunocompromised patient who suddenly deteriorates. And more over, delay or inability to diagnose strongyloidiasis frequently results in death despite treatment.

Hyper infection and disseminated strongyloidiasis require treatment for at least 7 days or until the parasite can no longer be identified in clinical specimens. Anthelminthic agents like benzimidazoles (thiabendazole, mebendazole and albendazole) disrupt energy production in the parasite as a result of disruption of cytoplasmic microtubule formation, thus killing both adult parasites and sterilize the larvae and eggs [22]. Oral administration of Tab. Ivermectin 200 micrograms per Kg body weight gives a very good response. It inhibits the neurotransmission in the parasite and soon eradicates the parasite from the host [23].

## Conclusions

Infection by *Strongyloides stercoralis* is very common in the tropical and subtropical regions. Most of the patients are asymptomatic or paucisymptomatic. However, life-threatening forms in the form of hyper infection syndrome and disseminated infection can occur occasionally especially in immunocompromised individuals. In most of the patients, it is not diagnosed at all or diagnosed very late. A high index of suspicion is required to diagnose and treat.
